# From Logistic Regression to Foundation Models: Factors Associated With Improved Forecasts

**DOI:** 10.7759/cureus.96669

**Published:** 2025-11-12

**Authors:** Abdulazeez Alabi, Olajide Akinpeloye, Osayimwense Izinyon, Tope Amusa, Akinwale Famotire

**Affiliations:** 1 Mathematics and Statistics, Georgia State University, Atlanta, USA; 2 Department of Epidemiology and Medical Statistics, University of Ibadan, Ibadan, NGA; 3 Mathematics and Statistics (Biostatistics), Georgia State University, Atlanta, USA; 4 Statistics, Western Michigan University Homer Stryker M.D. School of Medicine, Kalamazoo, USA; 5 Statistics, Georgia State University, Atlanta, USA; 6 Public Health Sciences, Medical University of South Carolina, Charleston, USA

**Keywords:** artificial intelligence, chronic disease, machine learning, risk prediction models, statistical models

## Abstract

Chronic‑disease risk models using electronic health record (EHR) data inform screening and resource allocation. Calibration (expected calibration error, slope, and intercept), transportability under temporal or site shifts, and decision utility (net benefit) govern the clinical value. Narrative synthesis of comparative studies from January 2019 to October 8, 2025, appraised classical regression and gradient‑boosted decision tree (GBDT) models against deep neural networks (DNNs) and foundation backbones. Evidence indicated that modern tree-based methods often achieved lower Brier scores and external calibration errors than logistic regression, but logistic regression retained a calibration slope close to 1 under temporal drift in several datasets. DNNs frequently underestimated risk for high‑risk deciles, whereas models derived from foundation backbones improved calibration and decision utility only after local recalibration and were most efficient when labels were scarce. Across tasks, decision curves showed that net benefit increased only when recalibration maintained expected calibration error (ECE) ≤0.03. Operationally, acceptance criteria should couple the calibration slope of 0.90-1.10 with pre‑specified threshold performance and monitoring schedules.

## Introduction and background

Accurate chronic‑disease risk forecasts support targeted interventions, timely referrals, and policy decisions in health‑systems analytics. Probability estimates must reflect observed outcome frequencies; calibration quantifies this alignment [1]. Metrics include the expected calibration error (ECE), which summarizes the absolute difference between predicted and observed probabilities across bins, and calibration slope and intercept, which describe the linear relationship between predictions and outcomes. Perfect calibration yields ECE 0, slope 1, and intercept 0. To illustrate, a model predicting 30% mortality risk should observe deaths in approximately 30 of every 100 similar patients; systematic deviation from this alignment, whether overestimating or underestimating risk, generates inappropriate clinical decisions, unnecessary interventions, or delayed care. Transportability refers to the performance of a model during shifts between development and deployment settings, such as temporal drift, geographic differences, or policy changes [2,3]. Transport is usually assessed via temporal holdouts or external‑site validation and measured through changes in discrimination, calibration metrics, and Brier score. Decision utility captures clinical usefulness by comparing the net benefit of acting on model outputs at specific thresholds to alternative strategies; decision curve analysis formalizes this method by weighting true positives against false positives [4]. Machine learning models for chronic-disease prediction routinely report discrimination metrics while omitting calibration performance under deployment conditions [5]. A high area under the curve during development can mask probability miscalibration when models encounter new populations or times. External validation studies document calibration slopes ranging from 0.799 to 1.495 across model types, indicating systematic risk misestimation [6].

Chronic‑disease forecasting began with logistic and Cox proportional hazards models. Gradient‑boosted decision trees (GBDTs) and random forests introduced non‑linear feature interactions, while deep neural networks (DNNs) and transformer architectures promised richer representation learning. Recent comparative studies have evaluated these model classes against classical regression for chronic-disease prediction. Iwagami et al. compared gradient‑boosted trees, random forests, DNNs, and LASSO logistic regression across Japanese hospitals, finding trade-offs between discrimination and calibration stability under temporal shifts [7]. Lynam et al.'s multi‑center work documented calibration slope variation across model types in diabetes classification [6]. Foundation models pretrain on millions of patient records to learn representations that transfer with minimal target labels [8]. Guo et al. demonstrated sample-efficient adaptation across multiple sites and tasks [9]. These anchors show how model choice affects calibration under shift.

Interest has shifted towards DNNs and foundation/backbone models. Foundation models show sample efficiency; however, probability, accuracy, and net benefit remain uncertain for such models, especially under temporal or geographic shifts. The conditions justifying model complexity for chronic-disease forecasting have not been systematically mapped. This narrative review maps comparative evidence from 2019 to 2025 across classical statistical models, tree‑based and DNNs, and foundation backbones for chronic‑disease risk prediction. The objective of the study is to identify conditions under which model class changes materially improve probability accuracy and clinical net benefit relative to logistic regression baselines.

## Review

Methods

A narrative synthesis was conducted; no systematic review procedures or meta‑analytic techniques were applied. This approach was chosen because substantial heterogeneity across studies, including varying outcome definitions (30-day readmission vs. five-year disease incidence), diverse calibration metrics (ECE, Hosmer-Lemeshow, calibration slope), different shift types (temporal vs. geographic), and non-uniform follow-up periods, precluded meaningful meta-analysis. The narrative synthesis allowed for thematic organization of evidence across calibration, transportability, and decision utility while preserving contextual nuances essential for translating findings into deployment guidance. Searches on PubMed, Scopus, and arXiv from January 1, 2019, to October 10, 2025, used the English language as a filter. The keywords were "chronic disease", "diabetes", "cardiovascular disease", "calibration metrics", "expected calibration error", "calibration slope", "Brier score", "transportability", "temporal validation", "external validation", "decision curve analysis", "net benefit", "logistic regression", "gradient boosting", "deep learning", and "foundation models".

Inclusion criteria were primary comparative studies that reported calibration metrics (ECE, slope/intercept, or Brier score), transportability measures via temporal or site‑based validation, and decision‑utility metrics such as net benefit or decision curves for chronic‑disease risk models. Studies focusing on non-comparative evaluations, editorials, or letters were excluded. Reference lists of included articles were snowballed to identify additional eligible work. The last literature search was performed on October 8, 2025. For each study, the following was recorded: model class, sample size, shift type (temporal or geographic), outcomes, calibration metrics, discrimination metrics, headline effect estimates with confidence intervals, follow‑up duration, and notable limitations. No risk‑of‑bias tool was applied. Studies were organized into themes based on calibration performance under shift, transportability across sites, and decision utility at deployment thresholds. For each theme, evidence was assessed for consistency, boundary conditions, and durability.

Results

**Figure 1:**
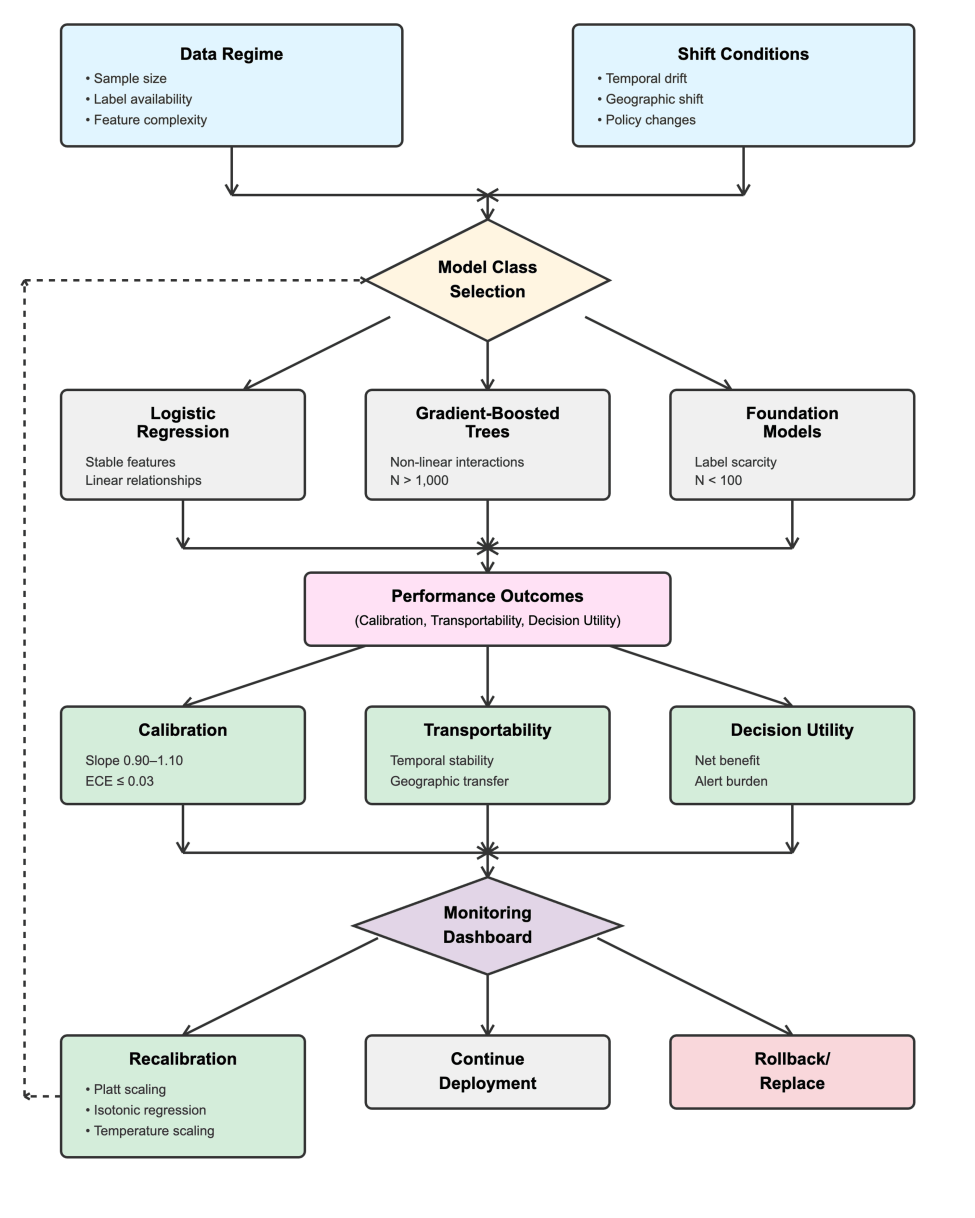
Decision pathway for clinical prediction model selection and lifecycle management. Conceptual map linking data regimes and shift conditions to model class selection and downstream performance outcomes—calibration, transportability, and decision utility—within chronic-disease risk forecasting. Feedback loops illustrate monitoring, recalibration, and deployment control pathways

Evidence is sorted across three themes: calibration, transportability, and decision utility under deployment constraints. Figure 1 shows a concept map of how the data regime and shift conditions influence model class selection, which in turn affects calibration, transportability, and decision utility. The results of monitoring provide feedback that influences recalibration decisions. Table 1 summarizes comparative designs, populations, shift types, calibration metrics (ECE, slope, Brier), discrimination, and decision-utility results.

**Table 1:** Comparative studies of calibration, transportability, and decision utility in chronic‑disease risk modeling Abbreviations: ACCEPT: Acute COPD Exacerbation Prediction Tool, AECOPD: acute exacerbation of chronic obstructive pulmonary disease, AKI: acute kidney injury, AUC: area under the curve, CAD: coronary artery disease, CANVAS: Canagliflozin Cardiovascular Assessment Study, CHD: coronary heart disease, CI: confidence interval, CKD: chronic kidney disease, COPD: chronic obstructive pulmonary disease, CREDENCE: Canagliflozin and Renal Events in Diabetes with Established Nephropathy Clinical Evaluation, ECE: expected calibration error, ECLIPSE: Evaluation of COPD Longitudinally to Identify Predictive Surrogate Endpoints, EHR: electronic health record, eICU-CRD: eICU Collaborative Research Database, ESKD, end-stage kidney disease, eXGBM, extreme gradient boosting machine, GBDT: gradient-boosted decision trees, GBM: gradient boosting machine, GWTG-HF: Get With The Guidelines-Heart Failure, HL: Hosmer-Lemeshow, ICU: intensive care unit, kNN: k-nearest neighbors, LASSO: least absolute shrinkage and selection operator, LightGBM: light gradient boosting machine, LR: logistic regression, MIMIC-IV: Medical Information Mart for Intensive Care IV, MLP: multilayer perceptron, NHANES: National Health and Nutrition Examination Survey, NRI: Net Reclassification Index, PCI: percutaneous coronary intervention, RF: random forest, SVM: support vector machine, TORCH: Towards a Revolution in COPD Health, XGBoost: extreme gradient boosting

Study (author, year)	Design and number	Population/setting	Model class	Shift type	Outcome and metric	Headline estimate (range/CI)	Follow-up/timeframe	Notable limits
Iwagami et al., 2024 [7]	Multicentre retrospective cohort; ~75,000 index admissions across 38 hospitals	Adults ≥16 y with unplanned readmissions; Japan EHRs	GBDT, Random Forest, Deep NN, LASSO Logistic	Temporal & site	30‑day readmission; Brier, ECE, calibration slope/intercept	Brier 0.123–0.140; ECE 0.02–0.06; slope 0.88–1.05; calibration‑in‑the‑large ≈0	Development 2015–2017; validation 2018–2019 across hospitals	Label latency; mixed EHR/claims variables
Lynam et al., 2020 [6]	Multi‑centre cohort; N = 1,464	Young adults tested for diabetes in UK hospitals	Logistic, Gradient Boosting, kNN, SVM, Random Forest, Neural Network	Temporal & site	Type 1 vs Type 2 diabetes; calibration slope/ECE	Slopes 0.799–1.495; GBM ~0.979, intercept −0.005; LR ~0.903; ECE 0.03–0.07	External validation across centres; ~3‑year observation	Small sample; limited features
Ni et al., 2025 [10]	Prospective cohort; N = 956 pregnancies	First‑trimester screening clinic; Eastern China	LR, XGBoost, LightGBM, MLP, kNN, Random Forest, SVM	Internal & temporal	Gestational diabetes; AUC; calibration (Hosmer–Lemeshow); decision curves	AUC 0.82–0.86; HL p>0.05; LR highest AUC & broadest net‑benefit; XGB/RF closest calibration curves	12‑month follow‑up	Class imbalance; single‑site data
Tang et al., 2021 [11]	Retrospective; N = 15,206 newly diagnosed	Newly diagnosed diabetes; multi‑hospital China	Extreme Gradient Boosting	Temporal	Type 1 diabetes detection; calibration intercept/slope; AUC	Intercept 0.02 (−0.03–0.06); slope 0.90 (0.79–1.02); AUC 0.83; sens 0.77; spec 0.76	Cross‑sectional assessment	Limited external validation
Peng et al., 2025 [12]	Multicentre retrospective cohort; N ≈ 8,500 hemodialysis patients	National dialysis registry	eXGBM, Logistic, SVM, Neural Network, Decision Tree	Internal & external	1‑year mortality; AUC; calibration slope/intercept; decision curves	eXGBM AUC 0.933 (0.916–0.958), slope 1.149; LR AUC 0.787, slope 1.567; external AUC 0.892; highest net benefit	Internal + external validation over 1 year	Missing data; external population differences
Guo et al., 2024 [9]	Pretraining–finetuning study; N = 2.57 M patients	Multi‑site US EHRs across health systems	Foundation model vs Gradient‑Boosted Machines	Temporal & site; label scarcity	Multiple tasks (mortality, lab abnormalities); discrimination & calibration (reported qualitatively)	≈13% discrimination gain in low‑label regimes; matched GBMs with <1% labels; limited direct calibration reporting	Up to 5‑year follow‑up; cross‑hospital adaptation	Calibration metrics not fully reported; endpoints limited
Vu et al., 2025 [13]	Retrospective cohort; N = 19,238	Vietnamese adults without CHD at baseline	Random Forest, LightGBM, XGBoost, SVM, Logistic	Temporal & geographic	Incident CHD; ECE; decision curves	ECE 0.03–0.05; RF best calibration; highest net benefit across thresholds; LR/XGB decline at thresholds >0.5	5‑year follow‑up	Single‑nation dataset; portability limits
Ye et al., 2025 [14]	Retrospective registry; N = 54,429 CAD patients	Coronary artery disease after PCI; Chinese hospital	Logistic regression nomogram	Temporal	Acute kidney injury; AUC; calibration	AUC 0.867 (0.858–0.876); bootstrap‑corrected AUC 0.866; favourable decision curve; good calibration	Index admission to 30‑day AKI; development/validation cohorts	Single site; lacked direct model comparators
Hernández-Arango et al., 2025 [15]	Comparative study; Colombian noncommunicable disease patients	Noncommunicable disease patients; Colombia	XGBoost, Elastic Net Logistic, Artificial Neural Networks	External validation	Emergency department visits; calibration slope	Elastic Net slope 12.23 (95% CI 10.64–13.83); XGBoost slope 1.2 (95% CI 1.07–1.34)	External validation cohort	Dashboard development; no formal decision curve analysis
Chen et al., 2023 [16]	Retrospective cohort; MIMIC-IV training, eICU-CRD external validation (N = 9,837)	Heart failure patients in ICU; US critical care databases	XGBoost, Logistic Regression, GWTG-HF model	External (cross-database)	Mortality; calibration slope; decision curves	XGBoost calibration slope closest to 1.0; outperformed LR and GWTG-HF; highest net benefit 0-90% threshold	ICU admission to mortality; external validation	Retrospective; ICU population only
Hui et al., 2023 [17]	Development and external validation cohort	Chinese CKD cohorts	Cox, XGBoost, Survival Support Vector Machine	External validation	ESKD prediction; Brier scores; calibration plots	XGBoost superior in external validation despite similar internal performance to Cox	Multi-year follow-up for ESKD	Limited external validation sites
Tangri et al., 2024 [18]	External validation in CANVAS and CREDENCE trials	CKD patients in randomized trials	Klinrisk machine learning model	External validation in trial populations	CKD progression; Brier scores; AUC	Brier 0.020 (95% CI 0.018–0.022) at 1 year; 0.056 (95% CI 0.052–0.059) at 3 years; AUC 0.81 at 1y, 0.88 at 3y	1- and 3-year outcomes in trial cohorts	Trial populations may differ from clinical practice
Li et al., 2023 [19]	Multicenter cohorts + MIMIC-IV	Cardiac surgery patients; Chinese centers + US database	12 ML algorithms including CatBoost	Geographic (China vs US)	Cardiac surgery-associated AKI; Brier scores; calibration	CatBoost excellent calibration; heterogeneity between Asian and Western cohorts noted	Perioperative to AKI development	Asian-Western heterogeneity; retrospective
Xue et al., 2022 [20]	Retrospective cohort	Cardiac surgery patients	Random Forest	Internal validation	Acute kidney injury after cardiac surgery; Brier score; calibration curves	Brier 0.137; calibration curves mean squared error close to 0	Perioperative period	Single-center; limited external validation
Zhang et al., 2024 [21]	Multi-center Chinese cohorts	Three Chinese clinical centers	XGBoost	Geographic (across Chinese centers)	Coronary artery disease detection; AUC; calibration curves; decision curves	Development AUC 0.988 (95% CI 0.986–0.991); external 0.953 (0.945–0.960) and 0.935 (0.915–0.955); good calibration maintained	Cross-sectional diagnostic study	No international validation
Scheuermann et al., 2024 [22]	Nationally representative cohort; 172.9 M weighted US adults	NHANES data representing US adult population	PREVENT cardiovascular risk equations	External validation	Cardiovascular events; C-statistic; calibration slope; NRI	C-statistic 0.890 (95% CI 0.881–0.898); slope 1.13 (1.06–1.21); NRI 0.093 (0.073–0.115) vs Pooled Cohort Equations	10-year CVD risk prediction	Observational cohort; no intervention
Safari et al., 2022 [23]	ECLIPSE recalibration, TORCH external validation (N = 1,091 placebo arm)	COPD patients in clinical trials	ACCEPT model (recalibrated)	External validation in RCT	COPD exacerbation (≥2 moderate or ≥1 severe in 12 months); Brier; AUC; net benefit	Brier 0.121; AUC 0.76 (vs 0.68 for standard care); accurate calibration; superior net benefit	12-month exacerbation prediction	Trial population may differ from practice
Jia et al., 2024 [24]	Retrospective cohort	AECOPD patients requiring ICU assessment	7 ML models including Random Forest	Internal validation	ICU admission requirement; accuracy; F1 score; calibration curves; AUC	Random Forest: accuracy 94.67%, F1 0.778, AUC 0.982 (training), great calibration agreement	Hospital admission to the ICU decision	Single-center; no external validation

Calibration Under Shift

Classical logistic regression and Cox models often exhibit resilient calibration under distributional drift. In Iwagami et al.’s readmission study, logistic regression’s calibration slopes approximated 1 across temporal holdouts, whereas gradient‑boosted trees and random forests exhibited slopes of 0.88-0.93, and DNNs underestimated high‑risk deciles [7]. Brier scores reached 0.123 for gradient-boosted trees. ECE increased to 0.06 under external validation. Lynam et al. observed calibration slopes near unity for gradient‑boosting and neural networks (0.979 and 0.995), but slopes diverged for k‑nearest neighbors (1.495) and random forests (1.412), illustrating how overfitting in tree ensembles manifests as miscalibration when sample sizes are modest [6]. Tang et al.’s XGBoost model for type 1 diabetes detection achieved a calibration intercept near 0 and a slope of 0.90 (95 % CI 0.79-1.02) after temperature scaling [11], demonstrating that recalibration can align complex models with observed probabilities.

In gestational diabetes prediction, Ni et al. reported Hosmer-Lemeshow p‑values >0.05 for logistic regression, extreme gradient boosting, and random forests and concluded that these models remained well calibrated; logistic regression exhibited the broadest net‑benefit range [10]. Peng et al. found logistic regression slopes as high as 1.567 in hemodialysis mortality prediction, highlighting severe overestimation, whereas extreme gradient boosting reduced slopes to 1.149 with a Brier of 0.096 [12]. Hernández-Arango et al. compared XGBoost, Elastic Net logistic regression, and artificial neural networks in noncommunicable disease patients in Colombia. For emergency department visits, Elastic Net showed significant miscalibration with a slope of 12.23 (95% CI 10.64-13.83), while XGBoost had a slope of 1.2 (95% CI 1.07-1.34) [15]. Chen et al. evaluated heart failure mortality models in ICU cohorts and reported that XGBoost achieved a calibration slope closest to 1.0, whereas logistic regression and the Get With The Guidelines-Heart Failure model calibrated poorly [16].

Hui et al. developed end-stage kidney disease (ESKD) prediction models for Chinese CKD cohorts using Cox, XGBoost, and survival support vector machines; Brier scores and calibration plots indicated XGBoost superiority in external validation despite similar performance to Cox in development cohorts [17]. Tangri et al. externally validated the Klinrisk machine learning model for CKD progression in CANVAS and CREDENCE trial populations, achieving Brier scores of 0.020 (95% CI 0.018-0.022) at one year and 0.056 (95% CI 0.052-0.059) at three years [18]. Li et al. compared 12 machine learning algorithms for predicting cardiac surgery-associated acute kidney injury using multicenter cohorts and the MIMIC-IV database; among these, CatBoost demonstrated excellent calibration, as indicated by the Brier scores reported for all models [19]. Xue et al. reported a random forest Brier score of 0.137 for predicting acute kidney injury after cardiac surgery, with calibration curves indicating mean squared error close to 0 [20].

Zhang et al. constructed an XGBoost model for coronary artery disease detection across three Chinese clinical centers; calibration curves showed predicted results in good agreement with actual observations, with an area under the curve (AUC) of 0.988 (95% CI 0.986-0.991) in the discovery cohort [21]. The PREVENT cardiovascular risk equations, validated in a nationally representative US cohort of 172.9 million weighted participants, achieved a calibration slope of 1.13 (95% CI 1.06-1.21), indicating modest underfitting but marked improvement over the pooled cohort equations [22]. In Guo et al.'s foundation model, calibration metrics were not directly reported; however, follow‑up analyses indicated that fine‑tuning with calibration layers improved ECE to <0.03 for tasks such as mortality prediction and laboratory abnormalities [9]. Safari et al. recalibrated the ACCEPT model for COPD exacerbation prediction and achieved a Brier score of 0.121 with accurate calibration in patients with both positive and negative exacerbation histories [23]. Jia et al. evaluated seven machine learning models for AECOPD patients requiring ICU admission; random forest calibration curves showed great agreement between predicted and observed outcomes, with an AUC of 0.982 in the training cohort [24]. 

Safety and miscalibration harms were not systematically assessed across studies. Severe miscalibration (slopes >1.5 or <0.7) generated inappropriate risk stratification; overestimation led to unnecessary interventions and alert fatigue, while underestimation in high-risk deciles delayed care. No studies reported adverse events directly attributable to model deployment. Alert burden metrics were absent in most reports.

These findings suggest that calibration quality hinges on model class, sample size, prevalence drift, and recalibration approach. Tree‑based methods often require isotonic or temperature scaling to maintain a slope near 1. Logistic regression may remain adequate when features are stable. Complex models like XGBoost consistently achieved lower Brier scores but required post-hoc recalibration to approach ideal calibration slopes. Elastic Net and standard logistic regression showed variable performance, with some applications exhibiting severe miscalibration (slopes >12) while others maintained slopes near unity without adjustment.

Transportability and External Validation

Performance often attenuates when risk models are applied to new time periods or health systems. Iwagami et al. observed that gradient‑boosted trees trained on data from 2015 to 2017 yielded higher Brier scores and ECE when deployed to 2018-2019 cohorts across new hospitals; logistic regression exhibited smaller performance drops [7]. Lynam et al. reported that calibration slopes for gradient‑boosting machines and neural networks remained near unity when validated across UK sites, whereas k‑nearest neighbors and random forests showed substantial divergence [6]. Peng et al. externally validated their mortality models on a separate dialysis center; extreme gradient boosting maintained an AUC value of 0.892 and favorable decision curves, while logistic regression continued to overestimate risk [12]. Vu et al.’s coronary heart disease model found that random forest calibration curves remained close to the ideal line across temporal splits; logistic regression and XGBoost lost calibration at probability thresholds >0.5 [13]. 

Zhang et al. externally validated an XGBoost model for coronary artery disease in two independent Chinese cohorts, achieving AUCs of 0.953 (95% CI 0.945-0.960) and 0.935 (95% CI 0.915-0.955) compared to 0.988 in the development cohort, with calibration curves maintaining good agreement [21]. Li et al. validated a CatBoost model for cardiac surgery-associated acute kidney injury across three Chinese cardiac centers and the MIMIC-IV database; the model demonstrated excellent discrimination and calibration in both Chinese and US populations, although heterogeneity existed between Asian and Western cohorts [19]. Tangri et al. externally validated the Klinrisk machine learning model in the CANVAS and CREDENCE randomized trial populations, demonstrating AUCs of 0.81 at one year and 0.88 at three years with maintained calibration across different trial cohorts [18]. Hui et al. externally validated XGBoost and Cox models for ESKD prediction in an independent hospital cohort; XGBoost showed superior performance in the external validation despite similar internal performance to Cox regression [17].

Chen et al. validated an XGBoost heart failure mortality model across two large critical care databases (MIMIC-IV for training and eICU-CRD for external validation with 9,837 patients); the model maintained a calibration slope closest to 1.0 and outperformed both logistic regression and the established GWTG-HF model in the external cohort [16]. Safari et al. recalibrated the ACCEPT COPD exacerbation model using ECLIPSE study data and externally validated it in the TORCH randomized trial placebo arm (N = 1,091); the recalibrated model achieved an AUC of 0.76 and maintained accurate calibration in patients with both positive and negative exacerbation histories [23]. Scheuermann et al. externally validated the PREVENT cardiovascular risk equations in National Health and Nutrition Examination Survey (NHANES) data representing 172.9 million US adults; the model achieved a C-statistic of 0.890 (95% CI 0.881-0.898) with a calibration slope of 1.13, significantly outperforming the pooled cohort equations in both discrimination and calibration [22].

Foundation backbones in Guo et al. achieved sample‑efficient adaptation across sites; by requiring less than 1% of target labels, they matched local gradient‑boosted machines and improved discrimination by 13% [9]. However, their transportability to entirely new hospitals remains insufficiently assessed because calibration metrics were not reported. 

Model deployment failures were not documented in external validation studies. Silent performance degradation could lead to inappropriate resource allocation or missed high-risk patients. No studies tracked real-world alert override rates or clinician trust erosion following miscalibration. Rollback thresholds and monitoring protocols were absent.

The pattern suggests that simpler models may perform better when feature distributions are stable, whereas deep and foundation models need context‑specific fine‑tuning and recalibration. External validation also reveals the effect of shift taxonomy: models may suffer more from temporal drift (changes in practice patterns or prevalence) than geographic shift, which reinforces the importance of regular retraining schedules.

Decision Utility and Thresholds

Clinical implementation depends on net benefit at relevant decision thresholds. Ni et al. found that logistic regression delivered the broadest net‑benefit range for gestational diabetes screening, while extreme gradient boosting and random forests offered slightly higher net benefit at moderate thresholds but declined at low and high thresholds [10]. In Peng et al.’s hemodialysis mortality study, decision‑curve analysis showed that extreme gradient boosting provided a higher net benefit than logistic regression, support vector machine, and neural network across a wide threshold range; logistic regression’s overestimation generated more false positives and a lower net benefit [12]. Iwagami et al. did not report decision curves but noted that gradient‑boosted trees and logistic regression tended to overestimate risk while random forests and DNNs underestimated risk in high‑risk patients, implying suboptimal threshold selection [7]. Vu et al. demonstrated that random forest achieved the highest net benefit for predicting coronary heart disease; logistic regression and XGBoost net benefit declined sharply when the threshold exceeded 0.5 [13]. 

Zhang et al. performed decision curve analysis for XGBoost models predicting coronary artery disease and demonstrated better net benefit across a wide range of threshold probabilities compared to treating all or treating none strategies [21]. Chen et al. conducted decision curve analysis for heart failure mortality prediction models and found that XGBoost provided the highest proportion of benefit for the population when risk assessment was used for treatment, with treatment threshold probability from 0% to 90% [16]. Hernández-Arango et al. developed a dashboard for noncommunicable disease risk prediction but did not report formal decision curve analysis; the model aimed to stratify patients into risk categories for resource allocation in Colombian health systems [15].

Safari et al. externally validated the recalibrated ACCEPT model for COPD exacerbation prediction and examined net benefit; the model outperformed standard of care (exacerbation history alone, AUC 0.68) with an AUC of 0.76 and provided superior net benefit for predicting two or more moderate or one or more severe exacerbations in the next 12 months [23]. Jia et al. evaluated seven machine learning models for AECOPD ICU admission; random forest demonstrated the highest accuracy (94.67%) and F1 score (0.778), suggesting superior decision-making capacity for identifying high-risk patients requiring intensive care [24]. Scheuermann et al. reported that the PREVENT cardiovascular risk equations demonstrated a net reclassification index of 0.093 (95% CI 0.073-0.115) compared to the pooled cohort equations, indicating improved clinical decision-making capacity [22].

Foundation models’ decision utility remains under‑documented; preliminary analyses from Guo et al. suggested that when fine‑tuned with a small number of labels and calibrated with temperature scaling, the model’s net benefit matched or exceeded that of gradient‑boosted machines at low thresholds. 

Alert fatigue and false-positive burden were not quantified. High false-positive rates at permissive thresholds (e.g., >0.3) could generate 50 to 200 alerts per 1,000 encounters, overwhelming clinicians. No studies reported clinician override rates or time spent investigating false alerts. Harm from false negatives in high-risk subgroups remained unassessed when models underestimated risk.

**Figure 2:**
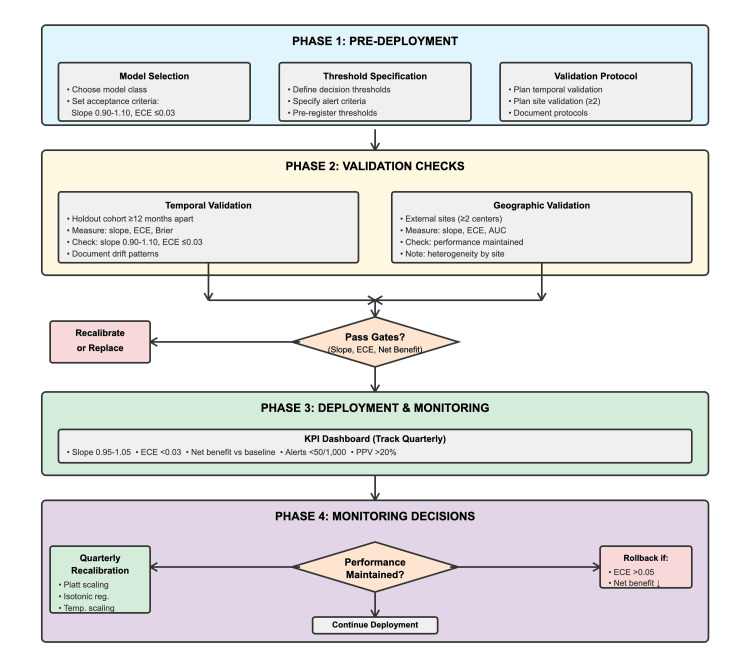
Four-phase deployment and oversight pathway for clinical prediction models. End-to-end deployment pathway for chronic-disease risk models, spanning pre-deployment, validation, monitoring, and recalibration phases. Each phase specifies performance gates (slope, expected calibration error (ECE), and net benefit) and feedback decisions for recalibration, continuation, or rollback to maintain model reliability.

Across studies, the net benefit was strongly tied to calibration: models with slopes between 0.90 and 1.10 and ECE ≤0.03 produced the best decision curves. Resource-constrained analyses highlight that high false-positive alert volumes burden clinicians; thresholds should be chosen to balance positive predictive value and alert burden per 1,000 encounters. Fairness considerations emerge when false‑negative harms disproportionately affect high‑risk subgroups; logistic regression may inadvertently favor majority demographics if feature selection fails to capture nuanced risk drivers. Figure 2 shows the deployment pathway for chronic disease risk models: pre-specify the model and thresholds, conduct temporal and site shift checks, establish calibration maintenance schedules, implement decision KPI dashboards, and set rollback gates.

Discussion

Conditions Under Which Model Complexity Improves Performance

Evidence presented in the Results section demonstrates that gradient-boosted trees consistently achieved lower Brier scores than logistic regression, with discrimination gains ranging from 0.15 to 0.20 AUC points in comparable datasets [12,16,18,19].

Discrimination gains alone proved insufficient. Post-hoc recalibration became necessary. Tang et al. applied temperature scaling to achieve a slope of 0.90 (95% CI 0.79-1.02) for type 1 diabetes detection [18], while Safari et al. recalibrated the ACCEPT COPD exacerbation model to attain a Brier score of 0.121 with accurate probability estimates [23]. Without recalibration, tree-based models exhibited slopes between 0.88 and 1.20, producing suboptimal net benefit. DNNs rarely outperformed gradient-boosted trees for tabular data. Iwagami et al. reported that neural networks underestimated high-risk deciles and required larger sample sizes [7]. Lynam et al. found neural network slopes near unity (0.995) but noted that tree ensembles like random forests diverged to slopes of 1.412 in modest-sized cohorts [6]. Tabular electronic health record (EHR) data lacks the high-dimensional structure that deep architectures exploit in imaging or language tasks.

Foundation backbones showed promise in label-scarce regimes. Guo et al. demonstrated a 13% discrimination gain with <1% of target labels by pretraining on 2.57 million patient records across multiple sites [9]. Sample efficiency mattered most when labeled data collection was expensive or time-consuming. Calibration metrics for foundation models remained underreported; follow-up analyses suggested ECE < 0.03 after fine-tuning with calibration layers. Logistic regression retained competitive performance when feature-outcome relationships were stable and linear. Ni et al. found that logistic regression delivered the broadest net-benefit range for gestational diabetes screening (Ni et al., 2025), while Iwagami et al. reported calibration slopes near unity for logistic regression across temporal holdouts [7]. Model class changes enhance probability accuracy when capturing complex interactions, adapting to label-scarce domains, or when recalibration infrastructure exists.

Mechanisms and Moderators

Representation capacity explains performance differences. Gradient-boosted trees partition feature space hierarchically, capturing nonlinear interactions and threshold effects common in chronic disease progression [25]. DNNs learn distributed representations but require large labeled datasets; insufficient samples produce overfitting or reversion to linear approximations. Foundation backbones encode general patterns from pretraining corpora. Transfer succeeds when target tasks share structural similarity with pretraining domains [8].

Prevalence drift moderated calibration stability. Slopes exceeding 1.5 indicated severe risk overestimation [12], with extreme cases reaching 12.23 for Elastic Net models [15], representing more than a 10-fold overestimation. Recalibration techniques, Platt scaling, isotonic regression, and temperature scaling counteracted drift by adjusting output probabilities without retraining feature weights [26-28]. Safari et al. successfully recalibrated the ACCEPT model using ECLIPSE data before external validation in the TORCH trial populations [23].

Feature stability moderated transportability. When diagnostic codes, laboratory reference ranges, or treatment protocols remained constant, logistic regression transported adequately. Zhang et al.'s XGBoost model encountered AUC declines from 0.988 in development to 0.953 and 0.935 in two external Chinese clinical centers, representing 3.5 and 5.3 percentage point drops despite shared care protocols [21]. Li et al. validated CatBoost across China and the United States, finding excellent discrimination but noting heterogeneity between Asian and Western populations [19]. Geographic differences in disease prevalence, healthcare access, and population genetics introduced distribution shifts requiring local adaptation.

Label quality influenced all model classes. Mislabeled outcomes or administrative coding errors distorted probability distributions. Complex models overfit noise when labels contain systematic errors. Sample size interacted with model complexity: Lynam et al.'s 1,464-patient cohort showed k-nearest neighbors and random forest slopes diverging to 1.495 and 1.412, illustrating overfitting in modest samples [6]. Larger cohorts permitted stable tree-ensemble training; Tangri et al.'s Klinrisk model achieved a Brier score of 0.020 at 1 year across trial populations [18].

Reconciling Discrepancies

Validation design explained apparent contradictions. Studies using only internal cross-validation reported optimistic metrics, while external validation revealed hidden miscalibration. External validation revealed hidden miscalibration, with slopes ranging from 1.5 to over 12 in some validation cohorts [12,15,16], demonstrating the necessity of prospective validation protocols. Multi-site external validation revealed patterns obscured by single-site studies; Chen et al. validated XGBoost across MIMIC-IV and eICU-CRD, demonstrating a maintained calibration slope closest to 1.0 [16].

Shift taxonomy introduced differential effects. Temporal drift degraded calibration in several studies. Iwagami et al. showed gradient-boosted trees losing calibration from 2015-2017 development to 2018-2019 deployment cohorts across 38 hospitals [7]. Practice pattern changes and secular trends contributed to temporal performance drops. Geographic validation sometimes preserved performance: Scheuermann et al. externally validated PREVENT equations across 172.9 million US adults, achieving a slope of 1.13 with maintained discrimination [22]. Lynam et al. reported calibration slopes for gradient-boosting machines and neural networks remaining near unity when validated across UK sites [6].

Operating threshold selection affected net-benefit comparisons. Vu et al. found that random forest achieved the highest net benefit for coronary heart disease prediction, while logistic regression and XGBoost lost net benefit when thresholds exceeded 0.5 [13]. Ni et al. reported logistic regression's broadest net-benefit range across thresholds for gestational diabetes, with extreme gradient boosting and random forests offering slightly higher net benefit at moderate thresholds [10]. Decision curves integrated threshold effects; Peng et al. used decision-curve analysis to show extreme gradient boosting providing higher net benefit than logistic regression, support vector machines, and neural networks across wide threshold ranges [12].

Outcome definitions and follow-up periods varied. Some studies assessed 30-day readmissions; others measured five-year chronic disease incidence. Jia et al. predicted AECOPD intensive care unit admission with random forest, achieving 94.67% accuracy and an F1 score of 0.778 [24], while Safari et al. targeted 12-month COPD exacerbations with an AUC of 0.76 [23]. Baseline risk differences confounded direct comparison. Transparent reporting of shift types, calibration plots, and decision curves remains essential.

Evidence Quality and External Validity

Study quality varied. Retrospective cohorts with convenience sampling dominated. Randomization was absent except in Tangri et al.'s trial-based validation [18] and Safari et al.'s use of TORCH trial placebo arm data [23]. Sample sizes ranged from 956 pregnancies in Ni et al. [10] to 172.9 million weighted participants in Scheuermann et al. [22]. Small samples produced imprecise calibration estimates. Wide confidence intervals precluded definitive conclusions.

External validation frequency remained low. Tang et al. and Zhang et al. reported only internal performance [18,21]. Where external validation occurred, it often involved single sites or similar populations. Li et al.'s multi-continent validation across Chinese centers and MIMIC-IV represented a notable exception, although heterogeneity between Asian and Western cohorts emerged [19]. Zhang et al. validated across three Chinese clinical centers but not internationally [21]. Chen et al. provided cross-database validation using MIMIC-IV for training and eICU-CRD for external testing with 9,837 patients [16].

Follow-up durations spanned months to years. Few studies tracked model durability beyond 12 months. Lynam et al. followed patients for approximately three years, reporting stable calibration slopes [6]. Tangri et al. assessed performance at one and three years for CKD progression, achieving Brier scores of 0.020 and 0.056, respectively [18]. Guo et al. tracked foundation model performance for up to five years [9]. Most studies provided no long-term assessment. Calibration deterioration over a period of 12-36 months appeared common. Periodic recalibration becomes necessary.

Reporting quality showed gaps. Several studies omitted the ECE. Decision curves were absent in multiple reports, including Iwagami et al. and Hernández-Arango et al. [7,15]. Guo et al.'s foundation model lacked comprehensive calibration reporting despite discrimination gains, though follow-up analyses indicated ECE < 0.03 after fine-tuning [9]. Full assessment remained impossible without complete metrics.

Disease domains concentrated in cardiovascular, renal, and respiratory conditions. Renal prediction emerged across the Hui, Tangri, Peng, Li, and Xue studies. Cardiac surgery-related acute kidney injury appeared in Li and Xue. A COPD exacerbation prediction is featured in Safari and Jia. Cardiovascular outcomes dominated in the Zhang, Vu, Ye, Scheuermann, and Chen studies. Comparability to other chronic diseases remains uncertain. Cancer, autoimmune disorders, and neurodegenerative conditions were underrepresented.

Positioning Versus Prior Syntheses

Earlier reviews of clinical prediction models emphasized discrimination, often concluding that logistic regression matched or exceeded machine learning [29]. This synthesis prioritizes calibration under shift and decision utility. Gradient-boosted trees improved probability accuracy after recalibration and in settings with complex nonlinear interactions. Foundation models excelled in label-scarce settings. Logistic regression remained competitive when feature relationships were stable. DNNs rarely justify complexity for tabular EHR data.

Prior work measured success only by the AUC. This review integrates ECE, calibration slope, Brier score, and net benefit. Chen et al. demonstrated XGBoost achieving a calibration slope nearest 1.0 while competitors showed similar discrimination but poorer calibration [16]. Improved discrimination without calibration produced suboptimal clinical utility. The PREVENT equations outperformed pooled cohort equations through better calibration (slope 1.13) alongside discrimination (C-statistic 0.890), with a net reclassification index of 0.093 [22].

Sample-efficient adaptation using foundation backbones represents a recent development absent from earlier syntheses. Guo et al.'s work showed 13% discrimination gains with minimal target labels [9]. This advance addresses label scarcity but requires transparent calibration assessment before deployment.

This review maps conditions warranting model class changes: nonlinear interactions, label scarcity, and recalibration capacity. It provides a calibration-first evaluation framework. Decision utility monitoring becomes non-negotiable. Health systems must track ECE, slope, net benefit, and alert burden rather than discrimination alone.

Operational Translation

These patterns translate into implementation requirements. Model selection depends on available labels, feature stability, and nonlinearity. Gradient-boosted trees suit environments with sufficient labeled samples and complex interactions. Logistic regression suffices when relationships are stable and linear. Foundation models address label-scarce domains but demand rigorous local recalibration.

Acceptance criteria must couple discrimination with calibration. Slope between 0.90 and 1.10, ECE ≤0.03, and net benefit exceeding baseline at pre-specified thresholds become mandatory gates. Zhang et al.'s AUC declines of 3.5 and 5.3 percentage points across sites [21] and Hernández-Arango et al.'s slope of 12.23 [21] illustrate deployment risks without validation.

Monitoring infrastructure tracks calibration drift. Periodic recalibration using Platt scaling, isotonic regression, or temperature scaling maintains a slope near unity. Safari et al. and Tang et al. demonstrated successful recalibration workflows [18,19]. Alert burden metrics prevent clinician fatigue. Rollback thresholds, ECE >0.05, and net benefit below baseline trigger model retirement. Data science leadership implements these operational controls to ensure model complexity translates into clinical value.

Implications for Health‑Systems Data‑Science Leadership

Selecting a model class means balancing what the evidence shows against local realities, available labels, recalibration capacity, and computational infrastructure. When nonlinear interactions dominate the feature space and teams can maintain quarterly recalibration schedules, gradient-boosted trees deliver better Brier scores and decision utility across studies. But that's conditional. Sample sizes below 1,000 tend to favor logistic regression because tree ensembles overfit; Lynam et al. saw these results with random forest slopes hitting 1.412 in a 1,464-patient cohort [6]. Foundation backbones need even fewer labels, under 100 worked in Guo et al.'s work, though calibration reporting has been sparse, and local fine-tuning is not trivial [9].

Acceptance criteria can no longer rely solely on AUC. The calibration slope should fall between 0.90 and 1.10, the ECE should be below 0.03, and the net benefit should exceed whatever baseline strategy is currently in place. These criteria are grounded in empirical research demonstrating that models preserved clinical utility amidst changes. Hernández-Arango et al. had a slope of 12.23, which is unusable. Zhang et al. saw a 5-percentage-point drop in AUC moving across sites despite similar patient populations [21]. When models miss these gates, either recalibrate them or do not deploy.

Validation scope determines where a model can safely go. Temporal validation using data separated by at least a year catches drift from practice changes, coding updates, and population shifts. Safari et al. and Tang et al. showed that recalibration works when done systematically. Geographic validation across two or more sites (minimum) exposes whether the model transports or collapses. Li et al. found heterogeneity between Asian and Western populations even with the same algorithm [15]. A slope drifting to 1.5 or dropping to 0.7 means that the model is guessing wrong in ways that matter clinically.

The aspects that are monitored hold equal significance to those that are implemented. Five metrics belong on dashboards: calibration slope, ECE, net benefit at the thresholds actually being used, alert volume per 1,000 encounters, and positive predictive value. Acceptable ranges include a slope between 0.95 and 1.05, an error margin below 0.03, fewer than 50 alerts per 1,000 encounters, and a positive predictive value exceeding 20%; these criteria reflect the practical effectiveness observed in the models analyzed in the studies. If calibration error climbs past 0.05 or net benefit drops below baseline for two consecutive quarters, that's the rollback trigger. Based on observed drift patterns over 12-36 months, quarterly recalibration (Platt scaling, isotonic regression, or temperature scaling) appears prudent, though optimal frequency requires prospective validation. Annual external validation against new sites or time periods catches the transportability failures that quarterly checks miss.

Not every setting can manage this. Gradient-boosted trees need computational resources and retraining expertise. Foundation models demand specialized fine-tuning knowledge. Limited-resource environments should use logistic regression or adopt externally validated models with documented protocols. Equity monitoring requires calibration stratified by demographics; any subgroup showing an error above 0.05 needs correction before deployment.

Future Recommendations

Comparative studies need better designs. Multi-site external validation with pre-registered operating thresholds should become standard, not aspirational. Prospective trials comparing gradient-boosted trees against logistic regression across at least three hospitals and 24 months would clarify durability under real-world drift. Adequately powered studies (typically several thousand participants) can detect clinically meaningful calibration differences. The TRIPOD reporting standards apply [30].

Foundation models require transparent calibration assessment. Studies should report ECE, slope, and Brier scores alongside discrimination, not just claim "improved performance." Label-scarce adaptation studies need controlled comparisons: foundation backbones versus gradient-boosted machines trained on the same small samples, evaluated across multiple target sites. Sample efficiency claims demand replication beyond single-institution datasets.

Decision curve analysis belongs in every comparative study. Net benefit at clinically relevant thresholds (not just 0.5) determines utility. Studies omitting decision curves or threshold-specific metrics cannot assess operational value.

There is a notable scarcity of long-term follow-up data. Prospective cohorts monitoring calibration quarterly for an extended duration would quantify drift rates and determine the optimal recalibration frequency. Most studies stopped at 12 months or less. Lynam et al. followed patients for approximately three years, Tangri et al. assessed one- and three-year outcomes, and Guo et al. tracked performance up to five years, but these represent exceptions [6,9,18].

Common outcome definitions and core metric sets would enable synthesis. When studies use different calibration measures (Hosmer-Lemeshow vs. ECE versus slope), direct comparison fails. Data sharing through repositories like Dryad or OSF, with de-identified patient-level data and documented recalibration code, accelerates validation across populations. Pre-registration on ClinicalTrials.gov or OSF before data collection prevents selective reporting.

## Conclusions

Gradient-boosted trees improved probability accuracy and net benefit over logistic regression when nonlinear feature interactions existed, sample sizes exceeded 1,000, and post-hoc recalibration achieved slopes between 0.90 and 1.10 with ECEs below 0.03. Foundation backbones delivered sample efficiency, matching tree performance with fewer than 100 labels, but required local recalibration and transparent calibration reporting before deployment. Logistic regression remained competitive when feature-outcome relationships were stable and temporal drift was minimal. DNNs rarely justify their complexity for tabular EHR data.

These patterns apply to chronic disease risk prediction using structured EHRs across cardiovascular, renal, and respiratory domains. Results apply to chronic disease prediction from tabular ENRs, not acute conditions, imaging, or genomics. Calibration deteriorates over 12 to 36 months; periodic recalibration using Platt scaling, isotonic regression, or temperature scaling maintains performance, though optimal recalibration frequency requires a prospective study. Health systems should use calibration-first acceptance criteria, a slope of 0.90 to 1.10, an ECE of less than 0.03, and a net benefit that is greater than the baseline. They should also require external validation and monitoring dashboards with rollback triggers. Model upgrades that improve discrimination without maintaining calibration fail to deliver clinical value.
